# Mucins Dynamics in Physiological and Pathological Conditions

**DOI:** 10.3390/ijms222413642

**Published:** 2021-12-20

**Authors:** Hassan Melhem, Daniel Regan-Komito, Jan Hendrik Niess

**Affiliations:** 1 Department of Biomedicine, University of Basel, 4031 Basel, Switzerland; 2 Roche Pharma Research & Early Development, Immunology, Infectious Diseases and Ophthalmology (I2O), Roche Innovation Center, 4031 Basel, Switzerland; daniel.regan-komito@roche.com; 3 University Center for Gastrointestinal and Liver Diseases, St. Clara Hospital and University Hospital of Basel, 4031 Basel, Switzerland

**Keywords:** goblet cell, mucus barrier, *Muc2* mucin, glycan, microbiota, inflammatory bowel diseases

## Abstract

Maintaining intestinal health requires clear segregation between epithelial cells and luminal microbes. The intestinal mucus layer, produced by goblet cells (GCs), is a key element in maintaining the functional protection of the epithelium. The importance of the gut mucus barrier is highlighted in mice lacking *Muc2*, the major form of secreted mucins. These mice show closer bacterial residence to epithelial cells, develop spontaneous colitis and became moribund when infected with the attaching and effacing pathogen, *Citrobacter rodentium*. Furthermore, numerous observations have associated GCs and mucus layer dysfunction to the pathogenesis of inflammatory bowel disease (IBD). However, the molecular mechanisms that regulate the physiology of GCs and the mucus layer remain obscured. In this review, we consider novel findings describing divergent functionality and expression profiles of GCs subtypes within intestinal crypts. We also discuss internal (host) and external (diets and bacteria) factors that modulate different aspects of the mucus layer as well as the contribution of an altered mucus barrier to the onset of IBD.

## 1. Introduction

The epithelial surface of our gastrointestinal tract (GI) is formed by a single layer of active cells that separates immune cells from an enormous source of stimuli. These include gut commensal bacteria, pathogens and food antigens [1]. Under normal conditions, immune reactions against the listed stimuli are well controlled, thanks to the mucus barrier. The barrier is formed by mucins, a large glycoprotein heavily decorated with O-linked glycans. The O-glycosylation process is of importance for mucin protection, as it creates a glycan coat that hides the protein core from protease degradation [2]. Mucins can be classified into transmembrane and gel-forming mucins [3]. It is important to note that *Muc2* is the major form of secreted mucins [4]. Differences in terms of thickness and type of mucins exist within the different segments of the GI tract. Numerous factors, such as cytokines, dietary, bacteria-derived factors and toxins, have been shown to modulate mucin turnover, including production, secretion and degradation processes, thus affecting the mucus barrier [5,6].

This review is divided in three subparts in which we will detail characteristics of the different GCs subpopulation, key factors regulating the biology of the mucus barrier and its implication in gut disorders.

## 2. Heterogeneous Population

**Figure 1:**
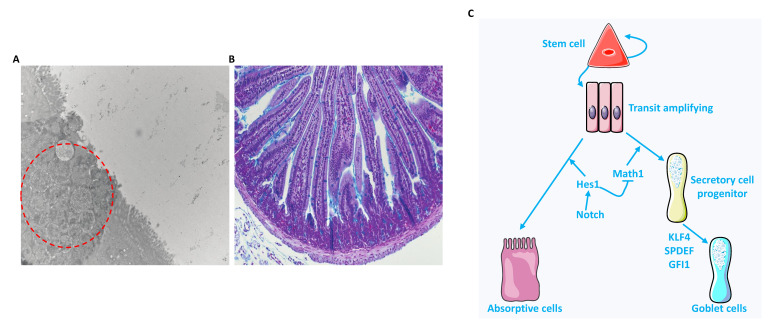
(**A**) Electron microscopy image taken from colonic murine section. (**B**) PAS Alcian Blue stained murine small intestine. Red circle indicates the mucin granules. (**C**) The intestinal stem cell differentiation into absorptive or secretory progenitor under the control of the Notch signaling. (Adapted from SERVIER MEDICAL ART (CC of license 3.0)).

Within the intestinal epithelium reside GCs, the primary secretory cells of the GI tract. They are recognized by their apical accumulation of mucin containing granules (Figure 1A) and they display positive staining to PAS Alcian Blue stain (Figure 1B). The production and secretion of mucins is the responsibility of GCs. Similarly to other intestinal epithelial subtypes, GCs derive by mitosis from Lgr5^+^ intestinal stem cells residing at the base of the crypt [7]. Transcriptional analysis has identified factors involved in GCs maturation and differentiation. For example, inhibition of the Notch pathway and its major effector protein Hes1 robustly drives progenitor lineage cells into differentiated GCs [8] (Figure 1C). Conversely, upregulation of Math1 (also known as Hath1) and Gfi1 promoted differentiation of progenitor cells into secretory cell lineages, including GCs. Further studies on the genetic regulation of GC maturation have identified Spdef as a key factor needed for proper GC maturation and function [9]. Classically, GCs are viewed as a homogenous secretory cell type. In light of new evidence, this view has changed, as a recent study indicates functional and gene expression profile differences of GCs in spatially distinct regions within the crypt. Transcriptional dysregulation of GCs marked by downregulation of WFDC2, an antiprotease molecule that preserves the integrity of tight junctions and prevents invasion by commensal bacteria and mucosal inflammation, was observed in IBD patients [10]. A recent study further demonstrated the diversity of GCs by identifying a subpopulation located at the colonic luminal, named intercrypt GCs (icGCs) [11]. Compared to the crypt-resident GCs, icGCs were characterized by a unique gene expression pattern and a different organization of mucus production with distinct properties [11]. Live tissue imaging revealed that icGCs mucus filled the regions between the mucus secreted by crypt-resident GCs and was impenetrable to bacteria-sized 1 μm beads [11]. The same study indicated fewer icGCs and disturbed mucus organization in biopsies from ulcerative colitis (UC) patients (both in active disease or in remission) [11]. In addition, a population of GCs, called goblet cell-associated antigen passages (GAPs), was shown to sense luminal antigens and deliver them to CD103^+^CD11c^+^ lamina propria dendritic cells [12]. These intraluminal antigens are subjected to mucus filtering whereby high molecular weight soluble antigens are excluded [12,13]. The preferential delivery to CD103^+^CD11c^+^ dendritic cells, which are suggested to induce tolerance to food antigens by interacting with regulatory T cells, implies an essential role of GAPs in intestinal immune homeostasis. Lastly, it has been shown that colon mucus includes a major layer produced by GCs of the proximal colon, which encapsulates fecal containing-bacteria, and a minor layer derived from GCs of the distal colon [14]. Together, these findings highly suggest that GCs subtypes within the intestinal epithelium are heterogenous.

## 3. Mucins

### 3.1. Classifications, Structural Organization and Differences within the GI Tract

Mucins are the major product of GCs. They form a biological gel covering the epithelium surface and are vital for maintaining gut homeostasis. This requires continuous secretion, estimated to be 10 L per day [15]. Water (90–95%), electrolytes, lipids (1–2%) and various proteins represent the key mucin elements [16,17]. To date, 21 different mucin genes have been identified [3]. Common to each mucin is a protein core characterized by a high frequency of the amino acids residues proline (Pro), threonine (Thr) and serine (Ser), named the PTS-rich domain [18]. Up to 80% of the mucin mass is made of O-glycans, indicating that the glycosylation represents a vital process for both protection and performance of mucin [6,19]. The mucins can be classified into two groups: transmembrane mucins and gel-forming mucins [3] (Table 1).

The transmembrane mucins include MUC1/3/4/12/13/15/17/20 and 21 [2,4]. Their main function is to protect enterocytes by covering their apical surface [20]. They vary in length with MUC16 being the longest and MUC13 being the shortest [3]. In the GI tract, transmembrane mucins are continually expressed with the exception for MUC1 and MUC16 [3].

The gel-forming mucins form the skeleton of the mucus system. They include MUC2/5AC/5B and 6, with MUC2 being the best characterized secreted mucin [3]. MUC5AC covers the stomach epithelial surface [21]. However, the mechanisms protecting MUC5AC from hydrochloric acid and pepsinogen in the stomach remain unknown. MUC6 is expressed by Brunner’s glands and duodenum [4].

**Figure 2:**
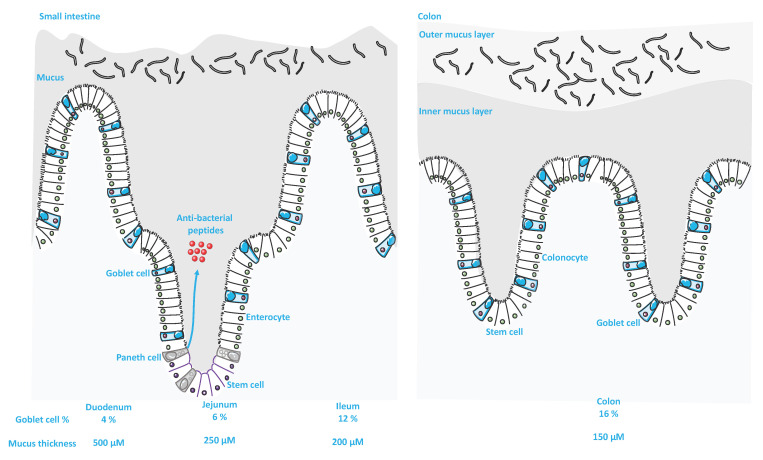
Differences between the small and large intestine. (Adapted from SERVIER MEDICAL ART (CC of license 3.0)).

Along the GI tract, GCs and the mucus layer differ in terms of distribution, composition, organization, thickness and expression profile (Figure 2). The percentage of GCs in the intestinal epithelium increases gradually from the duodenum (4%) toward the distal colon (16%) [22]. This abundance positively correlates with the distribution of intestinal flora distribution [5]. The small intestine displays a single nonattached mucus layer that is easily removed [23]. It is built around the mucin MUC2, which after secretion, remains anchored to GCs and requires the intervention of the protease meprinβ for its detachment [24]. The mucus layer in the small intestine is penetrable to bacteria; however, antibacterial mediators particularly those of Paneth cells fortify the mucus layer and keep away the bacteria from epithelial cells, except for segmented filamentous bacteria at the tip of the villi [6,25,26,27]. The enterocytes of the small intestine display the thickest mucus layer. In mice, an estimation of 500 μm, 250 μm, 200 μm and 150 μm thickness was shown for the duodenum, jejunum, ileum and colon, respectively [23].

Unlike the small intestine, the mucus layer of the large intestine consists of two layers: an outer and an inner layer. The outer layer is a nonattached, less dense layer and serves as a habitat for colonic bacteria. To the contrary, the inner layer is attached, continuously filled by the mucin MUC2 and almost free of bacteria [19,28].

### 3.2. Mucins and Intestinal Microbiota: Mutual Interaction

Mucin glycans serve as binding sites [29] as well as energy sources [30] required for the replication of microorganisms [6]. Through the activity of glycosidase enzymes, bacteria have the ability to degrade glycan [31]. These enzymes represent up to 40% of the bacterial genomes. Together, these observations clearly demonstrate a mutualistic interaction between the host and the gut microflora. On the other hand, the presence of intestinal microbiota is essential for GCs and the mucus layer. An elegant study [32] has investigated the physiology of the mucus barrier in two different wild-type C57BL/6 mouse colonies housed and bred separately in the same specific pathogen-free mouse facility. Surprisingly, one of the colonies had an impenetrable mucus layer whereas the other colony had an inner mucus layer penetrable to bacteria and beads [32]. This phenotype was confirmed by colonizing germ-free mice with the flora from the two colonies. Interestingly, a 16S rRNA gene sequencing analysis indicated different bacterial compositions specific to each colony [32]. The germ-free inner mucus layer is penetrable by bacteria-sized beads and thinner compared to those of conventionally raised mice [33]. As mentioned above, the protease meprinβ, required for the release of mucus in the small intestine, seems to be controlled by the presence of bacteria, as germ free mice and and meprin β-deficient mice have an attached mucus layer [24]. Moreover, several bacterial metabolites are increasingly recognized for their effects on goblet cells. In particular, short chain fatty acids (SCFAs; mainly butyrate, propionate and acetate) stimulate mucins and gene expression of other GCs products, such as TFF3 [34,35,36,37]. Similarly, the tryptophan metabolite indoleacrylic acid produced by the commensal *Peptostreptococcus* species and the aryl hydrocarbon receptor (AhR) were shown to induce goblet cell differentiation and function in mice [38,39]. Furthermore, SCFAs increase MUC2 expression via AP-1 and acetylation/methylation of histones at the MUC2 promoter [40]. The signaling of SCFAs by G-protein-coupled receptors (GPRs) promotes goblet cell function [41].

## 4. Modulators of Goblet Cells and the Mucus Layer

A large number of internal (host) and external (pathogens, diet and xenobiotics) stimuli have been reported to regulate the physiology of goblet cells, but also all steps of the mucin lifecycle, rendering this regulation very complex. Thus, uncovering the molecular mechanisms modulating goblet cell functions and mucin turnover is of special importance.

### 4.1. Immunomodulation

**Figure 3:**
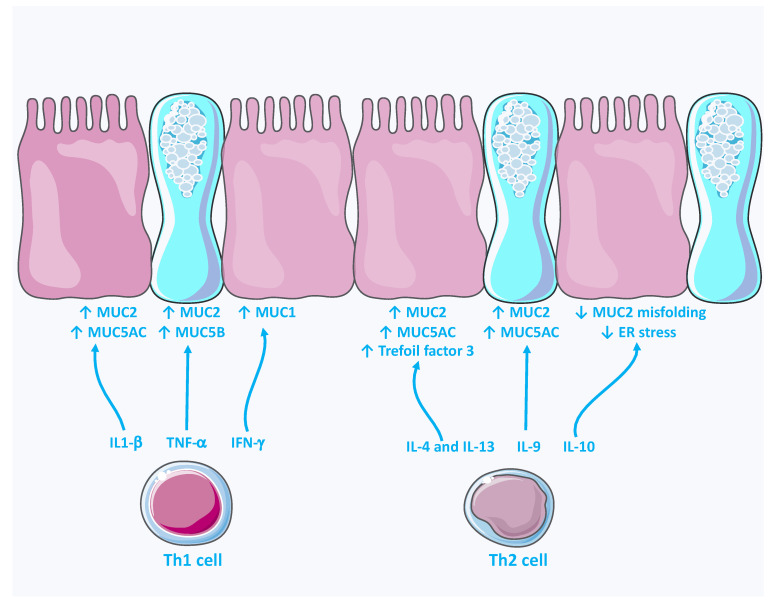
Mucin expression in response to type 1 and 2 cytokines. (Adapted from SERVIER MEDICAL ART (CC of license 3.0)).

Over the years, a series of studies showed that mucin expression/secretion is tightly controlled by bioactive cytokines. These cytokines are secreted by immune and non-immune activated cells. They are mainly classified into either type 1 (Th1; including IL-2, IL-12 and IFN-γ) or type 2 (Th2; including IL-4, IL-9, IL-10 and IL-13) cytokines. In general, the transcriptional regulation of mucins is mediated by the induction of signaling pathways, such as JAK/STAT, SAPK/JNK or MAPK, which in turn activate the transcription factor NF-κB [42,43]. A specific binding site of NF-κB has been described on the mucin promotor. In Figure 3, we summarize the mucin expression in response to cytokines.

Many Th1 cytokines, notably IFN-γ, TNF-α and IL-1β, appear to regulate the production/exocytosis of both transmembrane and gel-forming mucins. One study reported that IFN-γ positively modulates mucin exocytosis in the human colonic goblet cell line Cl.16E without altering mucin gene expression [44]. Another study reported that activation of IFN-γ receptor resulted in an elevated MUC1 expression level via the JAK/STAT pathway [45]. Similarly, TNF-α upregulated the expression of MUC2 and MUC5B but had no effect on the expression of MUC5AC and MUC6 in LS180 cells [46]. These findings were confirmed at the protein level [46]. The authors of this study also demonstrated that IL-1β stimulated the mRNA expression of MUC2 and MUC5A [46].

Furthermore, type 2 cytokines seem to strongly regulate mucin gene expression. Indeed, IL-4 and IL-13 upregulated MUC2 and MUC5AC transcription by activating either the STAT6 or NF-κB pathways [47,48]. Moreover, in HT-29CL.16E and HT29 cell lines, these cytokines also upregulated the expression level of other goblet cell products, such as TFF3 in a STAT-6-dependent manner [49]. In contrast, IL-4 and IL-13 displayed a negative regulation of mucin genes in non-colonic cell lines. For example, in nasal epithelial cells, IL-13 suppresses MUC5AC gene expression and mucin secretion [50]. In addition, the stimulation of human bronchial epithelial cells with IL-4 inhibited mucus secretion and downregulated the gene expression level of MUC5AC and MUC5B [51]. Another type 2 cytokine, IL-9, also induces expression of MUC2 and MUC5AC in human primary lung cultures and in the human NCI-H292 cell line [52]. Similar results were observed in C57BL/6J mice after IL-9 intratracheal instillation [52]. Furthermore, treatment of mice with anti-IL-10 resulted in the accumulation of unfolded MUC2 [53]. This effect was reversed by stimulating LS174T cells with IL-10 following pre-treatment with an ER stress inducer, namely, tunicamycin [53]. Incubation of LS180 cells with IL-6 induced the expression of MUC2, MUC5B and MUC6 [46]. Lastly, an IL-22 treatment ameliorated intestinal inflammation by enhancing mucus production in a STAT3-dependant manner [54].

Beside cytokines, numerous studies showed that deficiency of several inflammasome proteins, including NLRP6, ASC, and caspase-1, mediated autophagy impairment in GCs that resulted in altered mucin granule exocytosis, leading to impaired colonic host-microbial interactions [41]. In contrast, it has been demonstrated that colonic inner mucus layer formation and function are independent of NLRP6 inflammasome activity [55]. A variety of studies concluded a contradictory role of the inflammasome substrate cytokine IL-18. On one hand, IL-18 was shown to regulate the production of anti-microbial peptides and shape the homeostasis of the microbiome, thereby avoiding the outgrowth of colitogenic microbiota [56]. On the other hand, Nowarski et al., demonstrated that epithelial IL-18 drives goblet cell depletion and prevents functional goblet cell maturation [57]. A more recent study claimed that IL-18 produced by the enteric nervous system is essential for protection against bacterial infection via goblet cell antimicrobial peptide production, indicating a divergent role of IL-18 in colitis [58]. Lastly, mice lacking the bacterial sensor NOD2 displayed a decreased *Muc2* expression level and a higher abnormal fused granule [59].

### 4.2. Dietary Modulation

Nutrition impacts mucus layer characteristics. This is predominantly addressed by feeding mice with specific regimes recapitulating human diets. Non-digestible fibers and polysaccharides are generally considered as energy sources for gut bacteria. In the absence of fibers, bacteria use mucus glycan metabolism to fulfill its energy demands, suggesting an association between diet and mucins [60]. Accordingly, low-fiber diet is associated with a thinner mucus layer [61,62,63]. In a gnotobiotic mouse model, dietary fiber deficiency induced damage in the mucus layer, decreased the production of MUC2 and promoted the enrichment of mucin-degrading bacteria [64]. Together, these observations resulted in enhanced susceptibility to *Citrobacter rodentium* infection [64]. On the other hand, diets enriched in inulin fiber or probiotic bifidobacterial were shown to protect mucus deterioration [65]. Furthermore, the combination of fructooligosaccharide and resistant starch upregulated MUC2 expression in rats [66].

Western diets rich in saturated fats have been reported to cause a marked change in the functionality of the mucus barrier. In this context, reduction in fucosylated and sulfated residuals in the goblet cells of villi was observed in mice fed with a high fat diet during 25 weeks [67]. Another study revealed that a prolonged high fat diet leads to ER and oxidative stress in goblet cells reducing the production and secretion of mucins needed for a protective barrier [68]. This phenotype was more enhanced in *Winnie* mice [68]. Similarly, mice fed with high fat/high sugar displayed a thinner mucus layer and increased intestinal permeability and changes in microbial communities [69]. These modifications enhanced the ability of adherent-invasive *E. coli* to colonize the gut mucosa [69]. In line with these observations, a more recent study demonstrated that a high fat/high sugar diet created an inflammatory environment in the gut characterized by an overrepresentation of pro-inflammatory bacteria, such as Proteobacteria, and a significant reduction of SCFA concentrations [70]. Furthermore, food additives, including emulsifiers, are classified as agents that disrupt the mucus-bacterial interactions. Confocal microscopy analysis of mice fed with either carboxymethylcellulose or polysorbate-80 via drinking water indicated a closer bacterial residence to the epithelium and increased levels of mucolytic bacteria, including *Ruminococcus gnavus* [71]. These findings correlated with reduced mucus thickness without changes in MUC2 expression [71]. Moreover, the administration of maltodextrin promoted an activated ER stress in goblet cells and reduced MUC2 expression [72]. No significant change in mucosa-associated bacteria was observed in these mice [72].

Beside dietary fiber and the Western diet, a high-protein diet is associated with hyperplasic goblet cells and reduced mucosal myeloperoxidase activity [73]. A long-term high protein diet was shown to alter microbial composition in a rat model [74,75]. However, a short-term high protein diet had no significant effects on the gut microbiota composition [76].

## 5. Other Modulators

Delivery of granule mucin contents can be also induced by intracellular Ca^2+^ mobilizing agents. Among them, acetylcholine has been shown to stimulate mucin secretion in the small and large intestine. In addition, PGE2 promoted mucin secretion in a cAMP-dependent manner. Phorbol 12-myristate 13-acetate (PMA) also stimulated mucin release, implicating a responsive protein-kinase C-dependent pathway.

Xenobiotics, including aflatoxin M1, ochratoxin A, trichothecene mycotoxins toxin and deoxynivalenol, have been shown to affect goblet cell functions and MUC2 expression. This transcriptional inhibition was mediated by different pathways, including IRE1/XBP1, protein kinase R and mitogen-activated protein kinase p38 or mitochondrial dysfunction.

## 6. Bacterial Strategies for Overcoming the Mucus Layer

The continuity of the mucus layer is vital for intestinal homeostasis, as it forms the frontline defense of the gastrointestinal tract against bacterial infections [29]. Hence, bacteria need to contend with this barrier in order to access the epithelium. To this end, pathogens exploit several mechanisms allowing them to reach the epithelium in order to replicate and colonize.

Bacterial-derived products regulating mucin production and secretion:

### 6.1. Mucus Secretion

Induction. Several microbial products have been shown to regulate mucin transcription (Table 2). Among them, Gram-negative bacterial flagellin A binds to the surface receptor Asialo-GM1, resulting in ATP release [77]. This induces mucin transcription mediated by the phospholipase C/Ras pathway [77]. Similarly, lipopolysaccharide (LPS) form Gram-negative *Pseudomonas aeruginosa* and *E. coli* were described to upregulate MUC2, MUC5AC and MUC5B expression through the activation of the Ras-mitogen-activated protein kinase (MAPK)-mediated NF-κB activation pathway [78,79]. A number of bacterial species have been found to modulate GCs functions. Of these species, *Bifidobacterium dentium* [80] *Ruminococcus gnavus* [81] and *Lactobacillus rhamnosus* [82] regulate both mucin production and other mucosal defense factors produced by goblet cells, such as TFF3 and RELM-B. Furthermore, supplementation of the probiotics *Lactobacillus casei GG*, the cocktail VSL#3 and *Lactobacillus acidophilus* induced MUC2 mucin gene expression in Caco-2 and HT29 cell lines [83,84]. In agreement with this, Gram-positive lipoteichoic acid (LTA), a component of the cell wall, induced MUC2 mRNA expression in the human epithelial cell line HM3 by binding to the platelet-activating factor receptor [85].

*Inhibition*. Some pathogens opt for downregulating mucin production as a strategy to disrupt the mucus barrier integrity and promote their survival. Particularly, *Helicobacter pylori* and *Clostridium difficile* infections are closely associated with disrupted mucin synthesis and loss of mucus coat continuity. In this context, immunohistochemistry and in situ hybridization analysis on tissue biopsies of *H. Pylori*-infected patients indicated a significant decrease in MUC5AC expression level [86]. Furthermore, a 20% decrease in mucus thickness and 18% reduction in the gel-forming polymeric mucin content of mucus were observed in *H. pylori* positive subjects [87]. Exposure of gastric mucosal cells to *H. Pylori* LPS led to disrupted mucin synthesis and enhanced apoptosis [88]. These observations involved the ERK and p38 mitogen-activated protein kinase pathways [88]. In vitro studies revealed that MUC5AC and MUC1 synthesis was inhibited when KATOIII cells were incubated with *H. Pylori* [89]. Similarly, infection of the mucus-secreting human cell line HT29-CL.16E with *H. Pylori* resulted in inhibition of mucus secretion [90]. *C. difficile* is a common enteric pathogen and is known to provoke tissue damage and intestinal fluid secretion by the release of two toxins: A and B. Toxin A, produced by *C. difficile*, has been shown to induce a severe proinflammatory response characterized by excessive neutrophil recruitment [91]. In addition, toxin A stimulated the release of intracellular calcium and chemotactic response in human granulocytes [92]. Interestingly, toxin A inhibited the stimulation of mucin exocytosis in a dose-dependent manner without altering the baseline mucin exocytosis from HT29-CI.16E cells [93].

### 6.2. Degradation

Prior to infection, bacteria must traverse the mucus barrier. To this end, pathogens or commensal bacteria have evolved a wide repertoire of enzymes that cleave to glycoproteins.

Henderson and collaborators [94] identified and characterized a secreted protein named Pic (protein involved in intestinal colonization), which is encoded on the chromosome of both *Enteroaggregative E. coli* and *Shigella flexeri* and belongs to the subfamily of the serine protease autotransporters. The same study demonstrated a rapid proteolytic activity against bovine and murine mucus but not hog gastric mucin [94]. Surprisingly, another study claimed that Pic induces hypersecretion of mucus accompanied by an increase in the number of mucus-containing goblet cells [95]. Experimental *Helicobacter suis* infection disrupted mucin composition and glycosylation, decreasing the amount of *H. suis-*binding glycan structures in the pig gastric mucus niche [96]. Other pathogens use a different family of mucus-degrading proteins. Enterotoxigenic *E. coli* is strongly associated with severe diarrhea-mediated death in young children [97,98]. A key virulence element of this pathogen is the secretion of YghJ, a secreted and cell-surface lipoprotein. This enzyme belongs to the mucin-binding metalloprotease M60-like peptidase [99]. It promotes access of ETEC to epithelial cells by cleaving mucins of the small intestine, including MUC2 and MUC3 [99]. Of note, several studies have supported the use of this antigen as a broadly protective vaccine against the pathogenic *E. coli* species [100]. Shiga-toxin encoding the *E. coli* O113:H21 strain uses the subtilase cytotoxin for mucin degradation [101]. Bacterial proteases from *V. cholerae*, such as TagA [102] and extracellular chitinase (ChiA2) [103], have been described as mucin glycoproteins degraders. *V. cholerae* re-uses the release sugar from mucins in order to promote its survival in the intestine [103]. The pathogenicity of *Entamoeba histolytica* relies on the secretion of the cysteine protease 5 (EhCP5) [104]. Southern blot analysis indicated that the EhCP5 gene is missing in the non-pathogenic *Entamoeba histolytica* [104]. The cysteine proteases secreted from the *Entamoeba histolytica* disrupt the mucin polymeric network by specifically cleaving the C-terminal domain at two sites, whereas the N-terminal domain was resistant to proteolysis [105].

Overall, an altered O-glycosylation profile has been shown to have a disastrous impact on the functionality of the mucus barrier, as evidenced in different animal models. For instance, mice lacking the core 3β1, 3-N-acetylglucosaminyltransferase (C3GnT), the key enzyme for the synthesis of core 3-derived O-glycans, displayed a reduction in the *Muc2* protein and increased permeability of the intestinal barrier [106]. Moreover, these mice were highly susceptible to DSS-induced colitis [106]. In addition, the loss of core 1-derived O-glycans in mice resulted in significantly reduced mucus thickness characterized by the loss of the “b1” layer of the mucus [14]. Hyposulfataemic NaS1 sulfate transporter null mice exhibited a worsened DSS-colitis score and developed systemic infections when challenged orally with *Campylobacter jejuni* [107].

## 7. Mucins and Gut Disease

**Figure 4:**
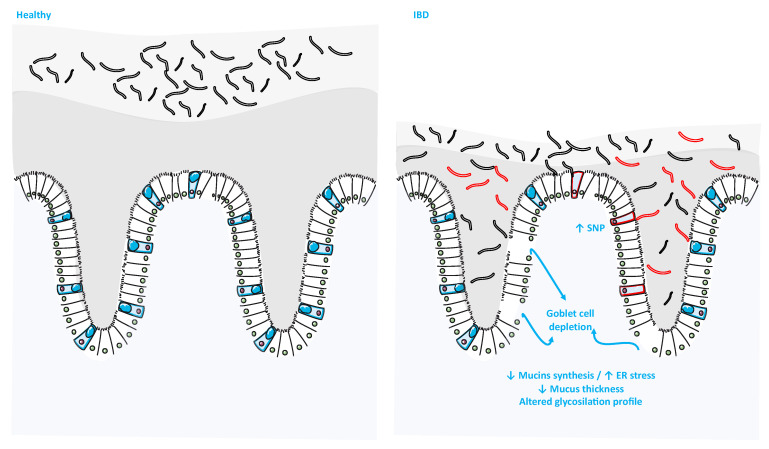
Goblet cells and the mucus layer in health and disease. (Adapted from SERVIER MEDICAL ART (CC of license 3.0)).

IBD, including Crohn’s disease (CD) and UC, is a chronic, disabling inflammatory condition of the gastrointestinal tract [108]. The peak of these disorders occurs during adolescence and early childhood [109]. The available therapeutic options for IBD are still unsatisfactory, which places a large burden on the health system. Thus, the development of new therapeutic strategies in the pathogenesis of IBD has become indispensable. One unique hallmark of IBD is an altered mucus barrier or of mucin production [110] (Figure 4). In support of this, several animal models carrying a disrupted barrier integrity displayed IBD-like syndromes [111,112]. An early meta-analysis of three combined genome-wide association studies has identified the MUC19 gene as a risk locus for IBD [113]. In CD, the existing evidence shows a significant discrepancy between the disease, GCs and mucus layer. Pullan and coworkers observed more mucus in CD patients [114]. Nonetheless, a more recent study indicated that the mucus layer and the level of GC stored mucin in CD patients were comparable to healthy controls [115]. In contrast, dysregulated MUC5AC, MUC5B and MUC7 gene expression was reported in CD patients [116]. Furthermore, MUC1 mRNA expression was significantly decreased in the involved ileal mucosa of CD patients when compared to the healthy mucosa [117]. An altered MUC2 structure was observed in CD patients, as indicated by shorter oligosaccharide chain length (50% less), leading to loss of its viscoelastic properties [118].

On the other hand, the relation between UC and the mucin layer is well established. UC patients display a thinner mucus layer as a result of the reduced GCs number and impaired MUC2 production and secretion [114,115]. MUC9 and MUC20 gene expression was found to be significantly decreased in patients with active UC compared to both remission group and normal controls [119,120]. Aberrant mucin *O*-glycosylation characterized by shorter glycans was observed in UC patients and is associated with increased inflammation [121].

On the animal level, genetic loss of *Muc2* leads to spontaneous colonic inflammation and higher susceptibility to DSS-induced colitis. In addition, Muc2^-/-^ mice display excessive bacterial contact with epithelial cells [122]. In contrast, Muc4^-/-^ mice demonstrated resistance to DSS-induce colitis, which can be explained by compensatory upregulation of the *Muc2* and *Muc3* level [123].

Another important candidate affecting the mucus barrier during IBD is the gut microbiota. The composition of intestinal microbiota is suspected to be a major risk factor for IBD pathogenesis. An increased abundance of mucin-degrading bacteria, including of the *Ruminococcus family*, has been reported in IBD patients [6,124].

Along with changes in gene expression, glycosylation or genetic polymorphisms associated with IBD, several cellular processes have been shown to be involved in the modulation of mucin secretion and to play a key role in the pathogenesis of IBD. GCs are one of the major epithelial cell types requiring robust endoplasmic reticulum (ER) function, which is due to the size, disulfide bonds and complexity of mucin transcripts, rendering them highly susceptible to inappropriate folding. During UC, GCs boost their mucin biosynthesis machinery to restore mucin expression, which can lead to an elevated level of misfolded mucin-induced ER stress. The use of Winnie and Eeyore strains demonstrated that misfolded *Muc2* resulted in abnormal GCs morphology, GCs apoptosis and the development of chronic intestinal inflammation [111]. In addition, the authors of this study reported an accumulation of MUC2 precursor that correlated with elevated levels of ER stress in the GCs of UC patients [111]. Two ER resident proteins named anterior gradient homolog 2 (AGR2) [125] and fatty acid synthase (FAS) [126] were shown to modulate the intestinal mucus barrier. AGR2 forms disulfide bonds with MUC2 on the A cysteine residue of its thioredoxin-like domain, suggesting a direct role for AGR2 in mucin processing [125]. Moreover, mice lacking AGR2 were viable but were highly susceptible to colitis [125]. FAS is an insulin-responsive enzyme essential for de novo lipogenesis. Genetic deficiency of FAS inhibited the generation of palmitoylated MUC2, profoundly damaging the mucus barrier integrity [126]. This results in increased intestinal permeability, higher susceptibility to colitis and dysbiosis [126].

ER stress has been linked to various pathways, particularly autophagy. It is defined as a vital catabolic cellular process through which host (misfolded proteins and damaged organelles) and bacterial-derived products are degraded via lysosomes [127]. In IBD, autophagy has been classified as a key element modulating the IBD pathogenesis [128]. Genetic ablation of autophagy proteins, including Atg54, Atg14 and FIP200, altered GCs exocytosis [129]. This study clearly demonstrates the significant influence of autophagy in modulating goblet cell function; however, the upstream signaling pathway that induces autophagy remains unknown.

## 8. Conclusions

The historic view of the mucus barrier as a static lubricant needed for food and stool progression in the intestine has evolved in favor of a highly dynamic organ as well as offering important protection against luminal microbes. Furthermore, the mucus layer actively contributes to the host–microbiota mutualism. Mucus layer impairment including altered expression, secretion, degradation, glycosylation profile and viscosity have been implicated in the pathogenies of IBD. A mounting number of stimuli are being discovered that could positively or negatively modulate these parameters rendering the regulation of GCs and the mucus layer a very complex system. Therefore, it remains unclear whether a disrupted mucus layer during IBD is a cause or consequence of inflammation. Thus, uncovering signaling pathways related to mucin biology could potentially facilitate the development of relevant treatments for chronic gut inflammatory disorders.

In this regard, primary attention should be given to answer the following questions:

What are the major chaperones needed for a proper mucin folding?

How bacterial metabolites modulate mucins?

Whether a cooperative bacterial work is needed to degrade the mucins?

How to target therapeutically a specific variant of GCs?

## Figures and Tables

**Table 1:** Human mucins type.

Type	Gene	Tissue Localization
Secreted	MUC5AC	Stomach
	MUC6	Stomach, Brunner gland and duodenum
	MUC2	Jejunum and colon
	MUC5B	Colon (weak expression)
Membrane	MUC1	Stomach, duodenum and colon
	MUC3A/B	Small intestine and colon
	MUC4	Stomach and colon
	MUC12	Stomach, small intestine and colon
	MUC13-15	Small intestine and colon
	MUC17	Stomach, duodenum and colon
	MUC20-21	Colon

**Table 2:** Modulation of mucin expression by bacterial components.

Species	Effector	Pathway	Mucin Expression–Secretion
*Pseudomonas aeruginosa*	Flagellin	ATP/Ca^2+^-ERK1/2	↑ MUC2
*Pseudomonas aeruginosa*	LPS	MAPK-NF-κB	↑ MUC2, MUC5AB, MUC5AC
*Bifidobacterium dentium*	-	Autophagy	↑ MUC2 and other GCs products
*Ruminococcus gnavus*	-	Peptide-like factors	↑ MUC2
*Lactobacillus rhamnosus*	-	-	↑ MUC2
*Staphylococcus aureus*	LTA	PAFR	↑ MUC2
*Helicobacter pylori*	LPS	PI3K/ERK MAPK	↓ MUC5AC, MUC1, ↓ secretion
*Clostridium difficile*	*C. difficile* toxin A	?	↓ secretion
